# Muscle Oxygenation Level Might Trigger the Regulation of Capillary Venous Blood Filling during Fatiguing Isometric Muscle Actions

**DOI:** 10.3390/diagnostics11111973

**Published:** 2021-10-23

**Authors:** Silas Dech, Frank N. Bittmann, Laura V. Schaefer

**Affiliations:** Devision of Regulative Physiology and Prevention, Department of Sports and Health Sciences, University of Potsdam, 14476 Potsdam, Germany; bittmann@uni-potsdam.de (F.N.B.); lschaefe@uni-potsdam.de (L.V.S.)

**Keywords:** muscle oxygen saturation, hemoglobin amount, isometric muscle action, O2C spectrophotometer, capillary recruitment, blood flow, holding isometric muscle action (HIMA), pulling isometric muscle action (PIMA)

## Abstract

The regulation of oxygen and blood supply during isometric muscle actions is still unclear. Recently, two behavioral types of oxygen saturation (SvO_2_) and relative hemoglobin amount (rHb) in venous microvessels were described during a fatiguing holding isometric muscle action (HIMA) (type I: nearly parallel behavior of SvO_2_ and rHb; type II: partly inverse behavior). The study aimed to ascertain an explanation of these two regulative behaviors. Twelve subjects performed one fatiguing HIMA trial with each arm by weight holding at 60% of the maximal voluntary isometric contraction (MVIC) in a 90° elbow flexion. Six subjects additionally executed one fatiguing PIMA trial by pulling on an immovable resistance with 60% of the MVIC with each side and same position. Both regulative types mentioned were found during HIMA (I: *n* = 7, II: *n* = 17) and PIMA (I: *n* = 3, II: *n* = 9). During the fatiguing measurements, rHb decreased initially and started to increase in type II at an average SvO_2_-level of 58.75 ± 2.14%. In type I, SvO_2_ never reached that specific value during loading. This might indicate the existence of a threshold around 59% which seems to trigger the increase in rHb and could explain the two behavioral types. An approach is discussed to meet the apparent incompatibility of an increased capillary blood filling (rHb) despite high intramuscular pressures which were found by other research groups during isometric muscle actions.

## 1. Introduction

During exercise, the demand for oxygen, nutrients and, therefore, blood increases in the muscular capillary system to transfer chemical energy into mechanical energy. During isometric muscle actions (IMAs), the regulation of blood filling of the microvessels is not entirely understood. In the past, a blood flow restriction or complete stop due to high intramuscular pressure have been discussed [1,2,3,4,5]. If the blood flow is restricted or stopped, the muscle ought to completely deoxygenate over time, i.e., the oxygen saturation decreases to zero. However, during IMAs maintained until muscle failure (“fatiguing measurements”), this assumption was not confirmed, at least in the examined superficial muscle layer [6,7,8,9,10,11,12,13]. On the one hand, the oxygen saturation showed an immediate decrease at the onset of loading [6,7,8,9,10] or short increase [11], followed by a leveling off into a steady state until fatigue [6,7,8,9,10,11]. These studies revealed that a homeostasis of oxygen delivery and consumption during IMAs is basically possible. On the other hand, a continuous decrease in the oxygen saturation with [12] or without [13] a previous slight increase has been described. However, it never decreased to zero until fatigue-related termination of the exercise. Other studies have limited the duration of muscle action [14,15,16,17,18,19,20,21] and, therefore, nothing can be said about the further progress of oxygen saturation. Nevertheless, steady states were also found in studies with limited loading durations [19,20,21].

Recently, our research group suggested to categorize the behavior of capillary venous oxygen saturation of hemoglobin (SvO_2_) and relative hemoglobin amount (rHb), as an indicator of the blood filling, roughly into two patterns (type I and type II) [6]. The crucial difference was the behavior of rHb. In type I, it ran nearly parallel to SvO_2_ (it only decreased and leveled off into a steady state), whereas in type II, it increased after reaching a reversal point (RP_1_). Subsequent to a leveling off into a steady state, rHb decreased in type II after termination of loading until a second reversal point (RP_2_) before returning to baseline level or higher. This behavior indicates a partial opposing behavior of both parameters [6]. The main objective of the present study was to clarify why rHb increases in type II. To investigate this, a closer look was taken at the course of rHb with regard to the behavior of SvO_2_ over time and in comparison of both types. A triggered regulatory mechanism by a boundary oxygenation level (threshold) was already hypothesized [6]. This should bring new insights into the regulative behavior of the microcirculation in the superficial muscle tissue during IMAs.

## 2. Materials and Methods

### 2.1. Subjects

Twelve Caucasian subjects (9 males, 3 females, mean age ± standard deviation (SD) = 29.75 ± 11.14 years) participated. Nobody had any health problems, to meet the one and only inclusion criterion. They weighed averagely 72.00 ± 11.03 kg and were 1.78 ± 0.08 m tall (BMI: 22.61 ± 1.93 $\frac{\mathrm{kg}}{m^{2}}$). Except for two left-handed subjects, everybody was right-handed. The study was conducted according to the declaration of Helsinki and was approved by the ethics committee of the University of Potsdam, Germany (approval No. 28-2017, 2 February 2018). All subjects were informed in detail and gave their written consent to participate.

### 2.2. Measuring Technique

To examine SvO_2_ and rHb of microvessels in the superficial muscle layers of the biceps brachii muscle, the O2C spectrophotometer was used (Oxygen To See; LEA© Medizintechnik GmbH, Gießen, Germany). The device operates with a combination of the laser Doppler technique and tissue spectrometry (laser light: near infrared, continuous wave, power > 30 mW; white light: 500–800 nm, 1 nm resolution). Previous studies have given a detailed description [22,23]. For a detection of SvO_2_ and rHb, only the spectrometry is relevant. The sent white light is backscattered in different wavelengths in dependence of the ratio of oxygenated and deoxygenated hemoglobin. The detected wavelengths are used for the calculation of SvO_2_ in %. The amount of light absorbed by the tissue is used for determining rHb in arbitrary units (AU). The specifications of the used flat probe (LF3, separation: 16 mm) allows for a light penetration depth of 12 mm. It was placed over the most prominent part of the biceps brachii muscle belly along its fibers. A double-sided adhesive film was used for fixation. The room light was dimmed to minimize light effects on the probe. The sampling rate was 40 Hz. The O2C device is valid and reliable [24,25,26] and is applicable to muscle tissue at rest [27] and during exercise [28].

### 2.3. Setting and Procedure

In the previous study, the differentiation of type I and type II was based on fatiguing measurements during a holding isometric muscle action of the left arm (HIMA) [6]. These measurements were also considered in the present study. The data set has been extended by measurements of the right arm of the same persons and two more subjects. Additionally, a fatiguing pulling isometric muscle action (PIMA) was performed by a subgroup (*n* = 6). The nomenclature of HIMA and PIMA was chosen according to Schaefer and Bittmann [29]. HIMA refers to an isometric muscle action while resisting an external force and was termed as “position task” [30,31,32] or “eccentrically loaded isometric contraction” [33] by other research groups. PIMA characterizes an isometric muscle action while force is developed against an immovable resistance in a pushing or pulling manner. This is also named “force task” [30,31,32] or “concentrically loaded isometric contraction” [33]. Both isometric tasks were performed in the present study since there are indications that these differ in various parameters [10,29,30,31,32,34,35,36]. However, there are also studies which did not find a difference between HIMA and PIMA [33,37,38].

**Figure 1:**
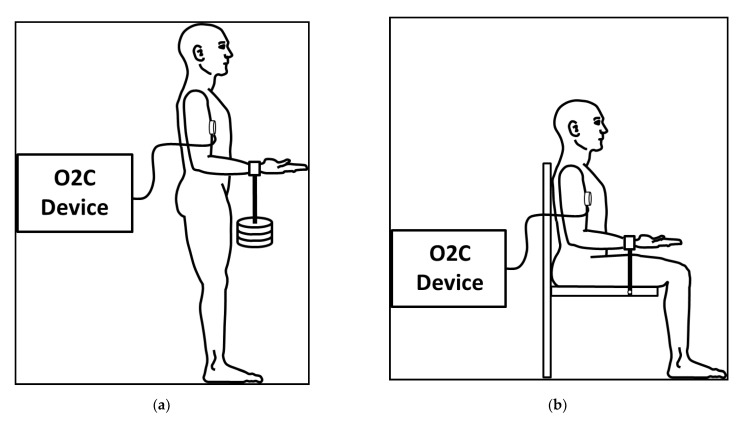
Measurement position and set up during a holding isometric muscle action (HIMA) (**a**), reprinted from Dech et al. (2020) [6] with permission, and during a pulling isometric muscle action (PIMA) (**b**).

All subjects performed a fatiguing HIMA trial, once with the right arm and once with the left arm. A subgroup of *n* = 6 additionally performed a fatiguing PIMA trial with each side. The settings are illustrated in Figure 1a,b. During all measurements, a cuff was applied 2–3 cm proximal to the wrist crease. The upper arm was in contact with the thorax, the elbow joint was flexed in 90° and the forearm was maximally supinated to emphasize the activity of the biceps brachii muscle. The intensity of every fatiguing trial was 60% of the individual maximal voluntary isometric contraction (MVIC). This intensity was chosen because it might be theoretically high enough to restrict the blood flow due to the intramuscular pressure [2]. Moreover, the loading times should be long enough to test for muscular endurance [39]. During HIMA, every subject had to hold a respective weight of 60% of the MVIC for as long as possible while standing. The weight was taken off as soon as the elbow angle exceeded 90° for 2 s, assessed by the rater subjectively. During PIMA, the participants sat upright and had to pull on a strain gauge which was connected to an immovable resistance. For maintaining the target force for as long as possible, subjects had visual feedback on a monitor. As soon as the force remained below the target force for 2 s, the rater prompted the subject to stop the task. The rest between every measurement was at least 3 min. The order of the arms performing the IMA was randomized. In addition, the order of tasks (HIMA or PIMA) of the subgroup was also randomized to minimize the effect of fatigue. The parameters (SvO_2_ and rHb) were recorded 10 s before every task in the measurements position and until 2 min of rest after loading.

To determine the MVIC, a HIMA or PIMA was performed in dependance of the fatiguing tasks (only HIMA or PIMA additionally). The arm and cuff positions were identical as already described. For the participants who only performed fatiguing HIMA trials, the MVIC was determined by the highest possible weight which could be held for 1 s in an upright standing position (Figure 1a). The weights were added progressively within maximal five steps and sufficient rest in between. For that, the rater hooked the respective weight onto the cuff and took it off after 1 s or if the elbow angle exceeded 90°. Because of the short duration, these measurements were not recorded by the O2C device.

The participants of the subgroup, who additionally performed fatiguing PIMA trials, sat in an upright position and pulled twice as strong as possible on a fixed strain gauge to record the maximum force (Figure 1b). These subjects were introduced to increase the force within 3 s to their maximum and hold it for at least 1 s. The rest between both trials was >2 min. These MVIC-tests were recorded by the O2C device.

The highest force value measured by the strain gauge (subgroup, maximal PIMA) or highest weight which could be held for 1 s (maximal HIMA), respectively, was determined as the MVIC.

### 2.4. Data Processing and Statistical Analysis

**Figure 2:**
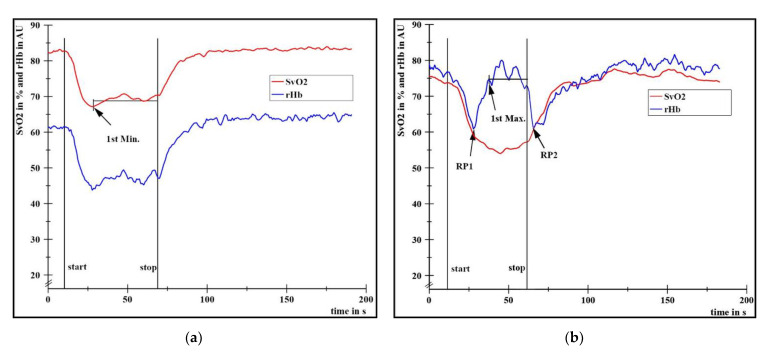
Curve examples of the capillary venous oxygen saturation of hemoglobin (SvO_2;_ red) and the relative hemoglobin amount (rHb, blue) in type I (**a**) with left arm and in type II (**b**) with the right arm of the same male left-hander (26 yrs, 1.85 m, 86 kg) during two fatiguing pulling isometric muscle actions at 60% of the MVIC of the biceps brachii muscle. Start and stop of loading are indicated by vertical lines. The first local minimum (1st Min.) was set exemplarily in (**a**) and the first local maximum (1st Max.) as well as reversal points (RP_1_, RP_2_) in (**b**). All curves were smoothed (moving average, maximal smoothing width: 50).

All curves were smoothed by using the software in NI DIAdem^TM^ 2017 (moving average, maximal smoothing width on one side: 50 points). With respect to the research question, the curves were categorized visually into type I or type II as described by Dech et al. (2020) [6]: type I: parallel behavior of SvO_2_ and rHb (Figure 2a); type II: partly inverse behavior of both parameters due to an increase in rHb after RP_1_ and decrease after stop of loading until RP_2_ (Figure 2b). The number of type I and type II measurements during fatiguing HIMA and PIMA trials as well as MVIC-tests were counted. Possible differences between HIMA and PIMA will not be considered here. This would be beyond the scope of the present study, i.e., to find a possible explanation of the two types. It will be presented within a more sophisticated study design (article in preparation). Without differentiation of HIMA and PIMA, the following variables were determined:

(1) Extent of SvO_2_ decrease (deoxygenation in type I and type II), calculated by the difference of the arithmetic mean (M) of the baseline values (initial 400 data points ≙ first 10 s) to the M of the plateau between the first local minimum (1st Min., Figure 2a) and stop of the task. Values of deoxygenation are presented in percent points (pp) and additionally in % related to the respective baseline value.
(2) Time to task failure (TTF), defined as the time period in s from start to end of loading.
(3) SvO_2_ levels at the moment of RP_1_ and RP_2_ (only in type II, Figure 2b), presented as Ms and 1.96-fold standard deviations (1.96SDs) in %.
(4) Time period in s from start until the minimum of rHb before start of its plateau (steady state). This corresponds to RP_1_ in type II.

IBM SPSS Statistics 26 was used for the statistical analysis. MVICs were compared between left and right arm as well as dominant and non-dominant arm. Differences of SvO_2_ baseline and deoxygenation levels (1) as well as TTFs (2) were compared between type I and type II. SvO_2_ levels at the moment of RP_1_ and RP_2_ (3) were analyzed to collect information about the main research question (existence of a threshold). In this regard, it was tested if differences exist between RP_1_ and RP_2_. All data were normally distributed (Shapiro–Wilk test, *p* > 0.05), except for three variables (fatiguing measurements: SvO_2_ baseline level of type I, SvO_2_ deoxygenation of type II; MVIC-tests: SvO_2_ level at RP_2_). Regarding normal distributed variables, analyses of differences were made by parametric tests (*t*-tests, variance homogeneity). Comparisons including one of the three not normally distributed variables were made by Mann–Whitney U test (independent samples) or exact Wilcoxon signed-rank test (dependent samples). Effect sizes are given for significant results (Pearson’s *r*):

(1) $$r=\frac{Z}{\sqrt{N}}\;\mathrm{or}\;r=\sqrt{\frac{t²}{t^{2}+df}}.$$

An alpha error of 5% was chosen for all tests.

Furthermore, correlations of SvO_2_ and rHb from start to the end of loading were determined by Spearman’s rank correlation coefficients (*ρ*) for every fatiguing measurement. Before calculating M ± SD for each type, Fisher’s *Z*-transformation was applied:

(2) $$Z=\frac{1}{2}\times \ln\;(\frac{1+\rho }{1-\rho })$$

M ± SD are presented after back transformation in *ρ:*

(3) $$\rho =\frac{e^{2Z}-1}{e^{2Z}+1}$$

## 3. Results

The averaged MVICs of all subjects did not differ significantly between the right (70.02 ± 23.83 Nm) and the left arm (69.31 ± 21.69 Nm, *t*(11) = 0.80, *p* = 0.442). This result did not change after correction for the lateral preference (dominant vs. non-dominant arm: *t*(11) = 0.57, *p* = 0.581).

### 3.1. Categorization of Measurements into Type I or II

Figure 2a,b illustrate the two behavioral patterns (type I and II) within the same subject during a fatiguing PIMA trial of the left and right arm. Based on the curve shapes, the visual categorization of all fatiguing measurements (HIMA and PIMA) as well as MVIC-tests (PIMA only) into type I (*n* = 25) and type II (*n* = 35) are given in Table 1. No measurement of the right arm during the fatiguing PIMA was assigned to type I. The categorization was not consistent within individuals because both types occurred in different trials in three female and three male subjects as shown exemplarily in Figure 2. During fatiguing HIMA trials of this subject, the behaviors were reversed for the left (type II) and right arm (type I).

### 3.2. Comparisons between Behavioral Types

Before the start of the fatiguing measurements, absolute SvO_2_ values in type I at baseline differed significantly (M ± SD = 78.04 ± 4.50%, *n* = 10) from type II (73.28 ± 5.97%, *n* = 26; *U* = −2.60, *p* = 0.008, *r* = 0.43). Before MVIC-tests, the difference between type I (71.37 ± 4.92%, *n* = 15) and type II (68.51 ± 4.34, *n* = 9) baseline values was not significant (*t*(22) = 1.44, *p* = 0.165).

During the fatiguing measurements of type I, SvO_2_ and rHb behaved nearly parallel to each other (Figure 2a) with a positive average rank correlation of *ρ* = 0.74 ± 0.61 (range: 0.19–0.99), *p* < 0.001. The minimum of rHb before leveling off into a steady state was reached after 11.41 ± 3.44 s on average. Subsequently to the end of loading, SvO_2_ approached to or increased above baseline value. In contrast, rHb decreased in type II until RP_1_ within averagely 6.85 s ± 3.39 s and approached to or increased above baseline value before leveling off into a steady state (Figure 2b). Consequently, the average rank correlation was negative (*ρ* = −0.81 ± 0.52 (range: −0.97–−0.18), *p* < 0.001). During recovery, rHb decreased until RP_2_ before increasing again to the baseline value or higher. In type II, the hemoglobin deoxygenated significantly more (–24.45 ± 11.59 pp ≙ –18.25 ± 9.35%, *n* = 26) than that of type I (–12.21 ± 3.67 pp ≙ –9.60 ± 3.09%, *n* = 10; *U* = −3.46, *p* < 0.001, *r* = 0.58). The TTF did not differ significantly between type I (45.88 ± 10.26 s) and type II (45.25 s ± 12.40 s); *t*(34) = 0.15, *p* = 0.886. All individual values of the presented variables during the fatiguing measurements can be found in the supplementary material (Tables S1 and S2).

During the MVIC-tests, the curve progresses of SvO_2_ and rHb were similar to the fatiguing measurements in respect to the regulative behavior (type I or type II). SvO_2_ also decreased significantly more in type II (–16.93 ± 3.41 pp ≙ –24.58 ± 4.12%, *n* = 9) compared to type I (–8.59 ± 3.55 pp ≙ –11.93 ± 4.64%, *n* = 15; *t*(22) = 5.66, *p* < 0.001, *r* = 0.77). However, SvO_2_ decreased until stop of the test or somewhat further before approaching to baseline value. Thus, no steady state was seen. Individual values of the MVIC-tests can be found in the supplementary material (Tables S3 and S4).

### 3.3. Oxygenation Level at Reversal Points

**Figure 3:**
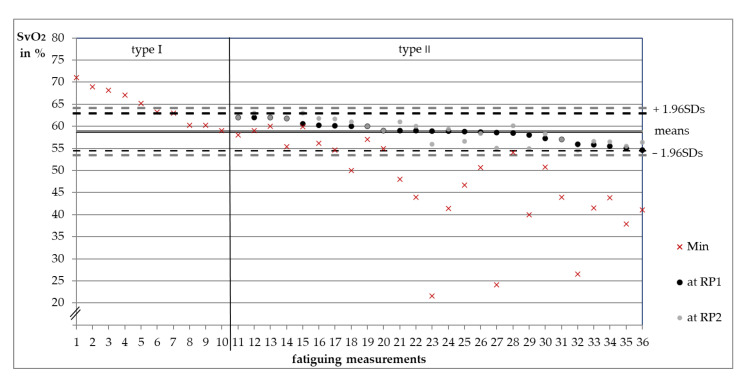
Capillary venous oxygen saturation of hemoglobin (SvO_2_ in %) of 36 fatiguing measurements of twelve subjects. Type I minimum values (red crosses, *n* = 10) sorted by oxygenation level. Type II SvO_2_ values at the first reversal points of the relative hemoglobin amount (RP_1_, *n* = 26, black, sorted by oxygenation level) and respective SvO_2_ values at RP_2_ (grey) as well as minimum values (red crosses). In some measurements, RP_1_ and RP_2_ are nearly identical (only grey dots). The horizontal lines express the arithmetic means of SvO_2_ values at RP_1_ (black, hidden) and RP_2_ (grey) of all 26 type II measurements. Dashed lines show the respective upper and lower 1.96-fold standard deviations.

**Figure 4:**
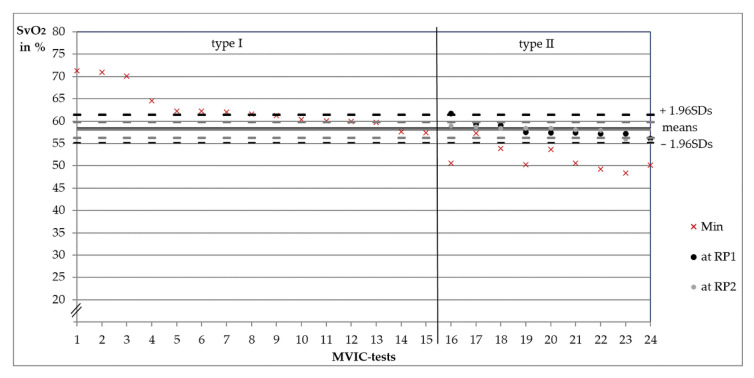
Capillary venous oxygen saturation of hemoglobin (SvO_2_ in %) of 24 MVIC-tests of six subjects. Type I minimum values (red crosses, *n* = 15) sorted by oxygenation level. Type II SvO_2_ values at the first reversal points of the relative hemoglobin amount (RP_1_, *n* = 9, black, sorted by oxygenation level) and respective SvO_2_ values at RP_2_ (grey) as well as minimum values (red crosses). The horizontal lines express the arithmetic means of SvO_2_ values at RP_1_ (black) and RP_2_ (grey) of all nine type II measurements. Dashed lines show the respective upper and lower 1.96-fold standard deviations.

According to the categorization of measurements, RPs of rHb exist only in type II. The SvO_2_ values at RP_1_ and RP_2_ of all type II fatiguing measurements are shown in Figure 3. The respective Ms and 1.96SDs are given. The SvO_2_ values between RP_1_ and RP_2_ differed not significantly (*t*(25) = –0.53, *p* = 0.600), whereby RP_2_ showed nearly the same Ms (58.91 ± 2.72%) as RP_1_ (58.75 ± 2.14%). During the MVIC-tests, SvO_2_ at RP_1_ was averagely 58.13 ± 1.66 pp and 57.82 ± 1.25 pp at RP_2_. Values were not significantly different (*z_exact_* = −0.53, *p* = 0.652, *n* = 9). Figure 4 shows M, 1.96SDs and individual values.

## 4. Discussion

Recently, our research group reported the occurrence of two regulative behaviors of oxygen saturation and blood filling in the venous microvessels (type I and type II) during a fatiguing HIMA at 60% of the MVIC and during an MVIC-test (PIMA) [6]. Type I showed a parallel behavior between SvO_2_ and rHb. In contrast, the main characteristic of the type II behavior is an increase in rHb while SvO_2_ decreases further on. The measurements of the presented study could also be clearly assigned to one of each type by visual inspection. Regarding the categorization of measurements in Table 1, type I and type II occurred during both isometric tasks. The finding that PIMA measurements of the right arm were only assigned to type II, is assumed to be due to the small sample size. This should be verified in future examinations. Furthermore, person and gender might not play a role because both types occurred within individuals and in males as well as in females.

During the fatiguing measurements at 60% of the MVIC, more type II than type I behaviors occurred. In contrast, during the MVIC-tests it was revers. The occurrence of more type I than type II behaviors in MVIC-tests might be a result of the loading duration. Our research group already argued that a maximal deoxygenation during short lasting MVIC-test (~4 s) might not be possible [6]. In fatiguing trials, the TTF was similar even though the hemoglobin in type II measurements deoxygenated more than these in type I. This result might indicate an independence of the behavioral types from the TTF during similar submaximal intensities and, therefore, possibly from the endurance capacity of the involved muscle. Thus, the onset of fatigue might not be explained by the level of deoxygenation. This is in accordance with Booghs et al. (2012) [10]. However, in the present study, the elbow angle was only controlled subjectively. Consequently, TTFs should be interpreted with care.

The main finding of the study was that during fatiguing measurements, rHb started to increase only in type II at an average SvO_2_-level of 58.75 ± 2.14%. In contrast, the SvO_2_ of type I measurements never reached that specific value during loading. This might be an indication of a SvO_2_-threshold around 59%, which seems to trigger the increase in rHb. Thus, dropping below the proposed threshold or not could explain the behavioral type. In the following, the discussion focuses on this potentially triggered regulation of microvascular blood filling and on the possible underlying physiological mechanisms behind it. Lastly, it is discussed how an increase in blood filling might be plausible during IMA, which are generally known for impeding capillary blood flow.

### 4.1. Triggered Regulation of the Capillary Venous Blood Filling

The two behaviors (type I and type II) might reflect a specific kind of regulation in dependance of the oxygen saturation level. An increase in the capillary venous blood filling (rHb) started at an average SvO_2_-level of 58.75%. The variation of that specific oxygenation level was very low (1.96SD = 4.16 pp), maybe indicating a specific and reproducible interpersonal threshold around 59%. A similar SvO_2_ level was found at RP_2_ (M = 58.91%, 1.96SD = 5.35 pp). RP_2_ occurred after stop of loading which might indicate an independence of such behavior from the muscular tension. In type I measurements, SvO_2_ partly reached but never decreased below 59%. Hence, the oxygenation level seems to determine the behavioral pattern (type I or type II). Reaching a SvO_2_-threshold of ~59% might trigger an increase in rHb. It is suggested that this threshold lies within a transition area (±1.96SD = ~55–~63%, Figure 3) which has to be passed to cause an increase in rHb in 95% of all cases. The hypothesized trigger mechanism might impede a further or complete deoxygenation and, therefore, might play an important role in regulating the blood filling of microvessels. According to these findings, the behavior of SvO_2_ and rHb might be parallel at saturation levels greater than ~59% and inverse until the saturation levels off into a steady state below that threshold. Consequently, rHb behaves parallel again but on a higher level. As SvO_2_ increases again after a stop in the loading but remains below ~59%, the behavior of both parameters is inverse and changes to a parallel one after exceeding the threshold again (Figure 2).

Other research groups also described or showed the progression over time of oxygen saturation, mostly expressed as tissue oxygenation index (TOI), as well as total hemoglobin (tHb), blood volume (BV) or total hemoglobin index (THI), measured by NIRS during fatiguing IMA [7,8,9,10,12,13,16,18,40,41]. This technique is comparable with the white light spectrometry used by the O2C device. Methodological differences have been discussed previously [6]. Most importantly, the O2C device detects microvessels < 100 µm in diameter and the NIRS technique vessels < 1 mm. Other studies have focused mainly on group level analyses [7,9,10,16,18] and/or presented only oxygenation data normalized to resting values [9,16,40,41]. This complicates a comparison with our approach, based on an analysis of rHb in relation to absolute SvO_2_ values of individual measurements. Booghs et al. (2012) [10], who analyzed the normalized THI of the biceps brachii muscle during a fatiguing isometric action at 60% of the MVIC, found a return of group mean THI towards the baseline values within the first 25% of the TTF. During this time period, the TOI dropped below 60%. Because of few presented time points (25%, 50%, 75% of TTF and immediately prior to failure) of the averaged data, it is not clear at which saturation level the normalized THI started to increase. In the study of Felici et al. (2009) [16], the grand average rest value of the TOI during a fatiguing IMA at 60% of the MVIC of the biceps brachii muscle was 69.5% ± 6.3%. According to their graphs, the tHb started to increase after the TOI dropped approximately 5–10 pp [16]. Although, these are only normalized mean values of seven subjects, this result might roughly fit our suggested threshold. Akima and Ando (2017) also presented graphical data [8]. The tHb of the quadriceps femoris muscle (all parts) increased on group level after the oxygen saturation reached about 60% [8]. Jones et al. (2014) [18] reported the mean values of the tissue saturation index (TSI) of the quadriceps femoris muscle during an IMA at 50% of the MVIC. These ranged from 58.5% to 59% [18], and these were partly below our proposed threshold (59%) but within the suggested transition area (~55–~63%). It might explain why they did not find an increase in the averaged tHb since type I measurements also reached the transition area in our data. In the study of Katayama et al. (2002), the TOI of the vastus lateralis muscle also did not decrease below the 59%-threshold on average during an IMA at 60% of the MVIC [9]. Their presented mean curve of tHb did not change significantly [9]. If some of their measurements showed only a decrease (type I) and others showed an increase above baseline values (type II behavior) (data not presented), changes in the mean tHb would level out. This might explain the insignificance. In a shown representative time course of original NIRS signals, the TOI decreased below 40% without an increase in the tHb [9]. This individual example does not support the presumed threshold. Furthermore, Moalla et al. (2006) examined the vastus lateralis muscle of healthy male children during an IMA of 50% of the MVIC [7]. They did not find an average increase in the BV, despite a drop of the average oxygen saturation below 20%. The reasons for the different findings could be very diverse, including, e.g., the used measurement technique, the examined muscles, the chosen intensity, fitness level and age of the participants. Because of the partly inconclusive results of heterogeneous studies, further research is necessary to validate the suggested ~59%-threshold in different muscles, fiber types and deeper regions as well as in younger and older persons. Moreover, in the presented study, only SvO_2_ and rHb and their possible dependance (trigger mechanism) were considered. Other variables such as “local increases in blood flow, temperature, carbon dioxide, acidity, adenosine, magnesium and potassium ions, and nitric oxide (NO) production” as listed by McArdle Katch and Katch (2010 [42], p. 334) could also be potential triggers in the regulation of blood filling. In addition, hydrogen ions, inorganic phosphate, prostaglandins and cytokines could be added to the list of potential substances [43]. In particular, the role of the radical NO will be addressed in the next subsection. However, the potential mediators might work synergistically instead of as one molecule alone [43].

### 4.2. Possible Physiological Explanation of the Regulative Response in Type II

As shown in the results and discussed before, in type II measurements, rHb initially decreased but increased after SvO_2_ reached 58.75% ± 2.14% on average. How could this increase in rHb be explained? One reason might be a stopped outflow due to a venous stasis [1]. However, this does not occur directly with the start of muscle action (initial decrease in type II). Furthermore, it would not be a general phenomenon at 60% of the MVIC, because in type I measurements rHb did not increase despite the same load intensity. In addition, different studies have shown that the oxygen saturation [6,7,8,9,10] and blood filling [7,44] decreased with a leveling off into a steady state during fatiguing IMAs. A venous stasis is not compatible with steady states if a complete anaerobic energy supply is excluded during fatiguing exercises. The steady states of SvO_2_ and rHb found in the presented data (type I and type II of fatiguing measurements) and other research groups imply a balanced delivery and consumption of oxygen and blood. Another reason for the increase in rHb could be a rise of cardiac output [12]. That is thought to be unlikely because only a small muscle group was activated in our study (arm flexors). Furthermore, the increase in rHb would be gradual instead of sudden as seen in the presented data. Thus, the increase found in rHb in type II could rather be the consequence of a redistribution of blood or red blood cells to the active motor units and/or of the increased blood flow in microcirculation [12,45,46,47,48]. This applies at least to the superficial muscle layer, where the intramuscular pressure is the lowest [2]. An accumulation of blood could be achieved by a local capillary recruitment [49]. Two recruitment theories have been described [50]. Both mechanisms behind the theories would expand the capillary O_2_-exchange area and have already been considered in analytical oxygen extraction models [50,51]. The older one hypothesized an opening of previously closed capillaries (binary recruitment) [50,52]. Despite a general acceptance in physiological and histological textbooks, the existence of precapillary sphincters as possible effectors is controversially discussed [43,48,53]. The scientific basis is limited to findings in the mesentery and could not be replicated in studies examining skeletal muscle tissue [53]. Furthermore, experimental and theoretical works challenge the hypothesis of closed capillaries during rest (for an overview see Poole et al., 2011 and 2020 [46,48]). In contrast, in the continuous or longitudinal recruitment theory a redistributed and homogenized blood flow of already perfused capillaries is hypothesized [46,47,48,50,54]. This approach considers the vasomotion of arterioles, the vessels with smoothed muscles, which regulate the blood flow. This stays in scientific consensus [53,54]. The arterioles also control the perfusion rate of the capillaries separately for red blood cells and plasma [43,48,54].

Thus, the increase in rHb could be the result of a vasodilation of arterioles. It is known that a vasodilation of blood vessels is initiated to maintain tissue oxygen consumption if systemic hypoxia arises [45,55,56,57]. In the regulation of the local vascular tone during exercise, the hypoxic induced release of NO seems to be the primary stimulus [56]. However, this concept remains controversial [55]. NO is inducted, e.g., by the neurohormone oxytocin [58], and it is regulated by β-adrenergic receptor mechanism during low-intensity exercises [56,57]. During higher intensities, vasodilation in skeletal muscle is clearly independent of this mechanism [56]. Thus, local dilatory mechanisms might be involved, but these are still not certainly identified [56,57]. In addition to systemic hypoxia, a local reduction in oxygen also stimulates vasodilation leading to a restoration of blood flow even though the compensatory response is not perfect [57]. The mechanisms behind this might be similar to systemic hypoxia [45,57]. Several pathways were suggested to explain the release of the vasodilatory NO. Adenosine or ATP release with activation of endothelial NO synthase, nitrate reduction by reaction of nitrate and deoxygenated hemoglobin as well as S-nitrosylated hemoglobin dependent bioactivity were discussed [55,56,57]. Further research is necessary to examine if such physiological mechanisms also apply to exercises including IMAs.

The blood filling in microvasculature might be embedded in complex regulatory processes to prevent a further deoxygenation of muscle tissue. A decreased oxygen saturation of hemoglobin is correlated with a local reduction in vascular resistance [45] and, therefore, vasodilation [59]. Thus, a SvO_2_ decrease (hypoxia) during muscle actions is assumed to be the stimulus in a negative feedback control system [45]. In this regard, SvO_2_ seems to be the controlled variable. The control of SvO_2_ might aim to achieve a homeostasis (reference condition) expressed as SvO_2_ steady states. The effector is the vascular smooth muscle in arterioles [45] which alters rHb via vasodilation or vasoconstriction. Thus, rHb would be the manipulated variable in such a loop regulation model. In contrast to the stimulus and effector, the sensor detecting SvO_2_-level (hypoxia) and the signals/activators regulating the vascular tone are not clear yet [43,45]. SvO_2_ or correlatives might be measured by sensors located in the endothelium, vascular smooth muscle cells, and red blood cells [56] or more precise in the hemoglobin [45]. The suggested threshold of ∼59% might be associated with a tipping point during accumulation of activators (metabolic messengers or rather endothelia factors as NO) leading to a vasodilation. This, in turn, leads to an increase in the rHb which is associated with an increase in oxygen delivery to meet the greater oxygen demand and preventing a further decrease in SvO_2_. Consequently, a steady state in SvO_2_ and rHb can be provided. According to the “bang-bang-theory” or “on/off-theory”, there might be an insensitive “dead-band” to the error signal (hypoxia) which enables a hysteresis of the blood filling [43]. This is necessary to prevent frequent adjustments in the vascular tone [43], and could explain the delay of blood support relative to the decrease in tissue saturation. The ∼59%-threshold could refer to the lower boundary of the “dead-band”. In comparison to regulations of other homeostatic systems, such an on/off behavior is normally not seen.

During muscle actions, the role of glucose should be considered in conjunction with oxygen [43]. Thus, the delayed vasodilation or initial vasoconstriction (decrease in rHb) could also be explained by an activated anaerobic glycolysis accompanied by an increasing lactate and superoxide production by membrane NAD(P)H-oxidase (Nox) at the beginning [43]. The superoxide (O_2_^−^) blocks NO signals until mitochondria start their work (recycling the NADH to NAD^+^ and resulting decrease in superoxide) [43]. In this regard, it could be reasonable that the start of mitochondrial activity plays a role in the variability of the suggested threshold (transition area). Regardless of the interpretation of the presented data, it remains questionable how the rHb can increase during IMA at all.

### 4.3. Increased Capillary Perfusion during Isometric Muscle Actions

**Figure 5:**
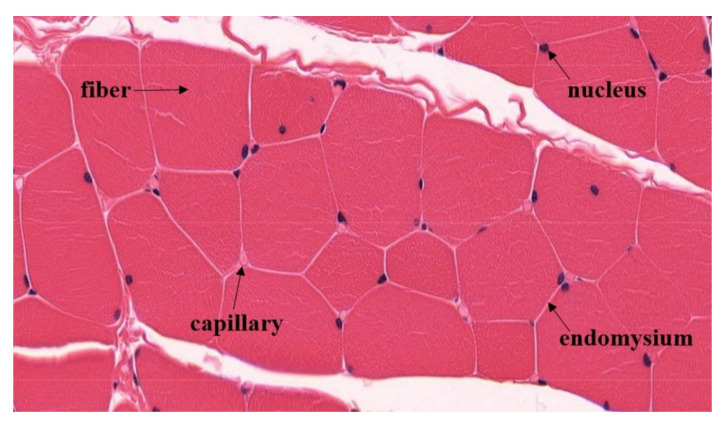
Cross-section of a striated human muscle. Capillaries are located in the endomysium where several muscle fibers adjoin. Cave: Nuclei (blue) are easily confused with capillaries (modified according to Sorenson and Brelje, University of Minnesota, Minneapolis, MN, USA, with kind permission [65]).

Isometric muscle actions at 60% of the MVIC, as performed in the present study, should lead to high intramuscular pressures resulting in a blood flow restriction or complete stop [1,2,3,4,5]. However, the present data and other experimental data showed that the blood filling can increase (type II behavior), even during MVIC-tests [6,8,16,41,60]. Although the MVIC-test of our presented data mainly showed type I behaviors without an increase in rHb (*n* = 15), there were also measurements which showed an increase (type II behavior, *n* = 9) even during maximal intensities of muscular work. A stopped outflow (venous stasis) as an explanation for the increase in blood filling seems to be unlikely, as already discussed in the section above. Thus, capillary blood flow in superficial muscle tissue is probably maintained during IMAs even at high intensities. A vasodilatory process as discussed in the previous section could be the primary mechanism to explain the increase in microvascular blood filling. To meet the apparent incompatibility, anatomical considerations regarding the location of the capillaries in muscle tissue could be an approach. To our best knowledge, this was not considered before. Most of the capillaries proceed parallel to the muscle fibers within the endomysium [61]. In a cross-sectional view, capillaries, not to be confused with nuclei [62], are primarily located in the endomysium where three or four muscle fibers adjoin each other [63,64] (Figure 5, taken from Brelje and Sorenson [65]). If muscle fibers contract or tense during a muscle action, their circumference will increase. According to the geometrical configuration [62,64], a widening of the triangular spaces in between seems to be reasonable. Thus, the whole blood filling capacity of capillaries would be available despite a greater muscular tension. Even negative pressure differences might arise thereby. Under the described anatomical circumstances, the intramuscular pressure could be higher than the capillary pressure. Moreover, Poole and colleagues argued that muscle capillaries do not collapse easily under conditions of increased muscle or reduced intraluminal pressure. They explain this by the presence of collagen struts [46,48]. Furthermore, nerve fibers are also located in the endomysium [64,65], and they might be protected from the muscular compression due to their anatomical position, too.

In conjunction with the anatomical considerations, the muscular blood flow during IMA might be supported by oscillations. Muscles oscillate transversally around 10–15 Hz during IMA [19,29,35,66,67,68,69,70,71]. These oscillations are an expression of the firing rate of motor units [19]. Thereby, the amplitude increases with higher intensities of muscle action [67]. Yoshitake et al. (2001) found a nearly constant group mean frequency of 10.5–11.9 Hz from start until the end of a fatiguing isometric back extension in the mechanomyographic signal (MMG) of the erector spinae muscles [19]. However, the amplitude in the MMG signal increased significantly at first and then decreased continuously until fatigue [19]. The absolute contraction intensity is not known in that study but is estimated to be 45% on average. However, the found behavior of MMG amplitude is not identical over all intensities during sustained contractions. It tends to increase at force levels between 10% and 40% of the MVIC, does not change or decreases from 50% to 80% and decreases from 90% to 100% [72]. Moreover, there are also indications of differences in the courses of MMG amplitudes between fiber types [73]. Yoshitake and colleagues (2001) discussed that the reduced amplitudes found in their examination resulted from slowed contractile elements (extension of relaxation time) and, therefore, decreased dimensional changes of the active fibers [19]. From their point of view, this would result in an increased intramuscular pressure and a restriction of the muscle blood flow, and could consequently explain the onset of fatigue. In contrast, MMG amplitude seems not to change with an increase in intramuscular pressure [74]. Moreover, due to maintenance of muscular oscillations until fatigue independently of the amplitudes, the opinion of a blood flow restriction per se might be challenged. Our research group already supposed the oscillatory behavior might serve as a pump and, therefore, could support the capillary blood flow [29]. In addition, Yoshitake et al. (2001) found steady states of the oxygenated hemoglobin amount after an initial decrease recorded together with the MMG [19]. As discussed before, a steady state implies a balanced oxygen delivery and consumption. If the blood flow is restricted while the demand increases in the exercising muscle, the available oxygen will be depleted over time. However, this has not been seen in experimental studies yet. Thus, the onset of fatigue seems not to be explained by the oxygen supply, but rather by other metabolic or neuromuscular factors.

### 4.4. Study Limitations

No gold standard exists for muscle oxygen saturation measurements [75]. The near infrared spectroscopy or white light spectrometry as used in the present study are recently used techniques. Limitations were described previously [6,76,77,78]. In brief, it should be noticed that an influence of arterial blood cannot be completely excluded in the recordings [76,77,78,79]. Regions of the muscle deeper than 12 mm with possible higher intramuscular pressures [2,80] cannot be examined by the device used in the present study. In this regard, it should be questioned if results would be different in deeper muscle layers. In a more advanced study design, the specific morphology of examined soft tissue, e.g. the individual muscle thickness, could be controlled by functional echomyography or computed tomography scans (CT-scans) in future measurements [81,82,83]. In this regard, the subcutaneous fat layer also plays a role and might have affected the measured parameters [84]. The skinfold thickness was not examined, but we assume low values in our normal weighted participants (BMI: 22.61 ± 1.93 $\frac{kg}{m^{2}}$) for whom the skinfold thickness above the examined biceps brachii muscle is regularly low. If the fat layer was too thick, the white light would not have reached muscle tissue and no change in the SvO_2_ would be recognized. However, the thickness of the fat layer could have been different and, thus, tissue penetration depths of the white light might have been different in our subjects. Furthermore, six MVICs were determined by gold standard (strain gauge, PIMA). The other six were determined by weight holding (HIMA) and could not reach the same accuracy. The influence is expected to be small. As already mentioned, the termination of loading during the fatiguing HIMA trials was not standardized. The examiner stopped the loading as soon as the elbow exceeded 90° for more than two seconds according to visual inspection. This might have influenced the TTF. At last, the small sample size and differences in the age (min.–max.: 19–58 yrs.) of the explorative study must be mentioned. Despite all limitations and very clear findings, it could be worthwhile to examine a greater sample including participants of different ages and different fitness conditions.

## 5. Conclusions

Based on the method used, the presented data highlighted the obvious relation of oxygenation and blood filling of microvessels in superficial muscle tissue. It was found that the blood filling (rHb) can increase after a previous decrease during IMA at intensities of 60% and even of 100% of the MVIC. This reversal seems to be related to the amount of deoxygenation. Based on the results it is assumed that the reversal occurs if the oxygenation level decreases considerably (transition area around 59% SvO_2_). It is hypothesized that this might trigger the regulation of blood filling.

The method used does not allow for direct conclusions on capillary blood flow, but indirect conclusions by regarding rHb and SvO_2_ behaviors can be drawn. The increase that occurred in blood filling and steady state of oxygen saturation indicate a maintained capillary blood flow and against a venous stasis, as was discussed. On the basis of these findings, we propose to reconsider and discuss the current concept of a principally restricted or stopped capillary blood flow during IMAs. Possible theoretical explanations for the maintenance of blood flow and for an expansion of the O_2_-exchange area (indicated by an increase in rHb) might be found in the special anatomical location of capillaries and the mechanical oscillations of muscle fibers during IMA. Due to those properties and features, the steady states found might have emerged. The mechanisms are not clear yet and, therefore, it is suggested to take a closer look at those anatomical and functional aspects in future studies. Further research is needed to investigate if deeper regions of muscles and different muscle fiber types show similar behavior. Furthermore, the possible influences of age, training and health status should be examined. Supposing that the findings hold true in future studies, deviations from regulative norms could potentially serve as an early diagnostic tool for metabolic disorders, myopathies or even chronic fatigue syndrome.

## Figures and Tables

**Table 1:** Number (*n*) of type I and type II behaviors of the capillary venous oxygen saturation and blood filling separated by task and arm of twelve subjects.

	HIMA		PIMA			
Task	Fatiguing		Fatiguing		MVIC-Test	
arm	left	right	left	right	left	right
type I (*n* = 25)	5	2	3	0	8	7
	7		3		15	
type II (*n* = 35)	7	10	3	6	4	5
	17		9		9	
total (*n* = 60)	12	12	6	6	12	12
	24		12		24	

## Data Availability

The data presented in this study are available in the main article and Supplementary Materials.

## Supplementary Materials

The following are available online at https://www.mdpi.com/article/10.3390/diagnostics11111973/s1, Table S1: Single values of fatiguing measurements of type I behavior. Table S2: Single values of fatiguing measurements of type II behavior. Table S3: Single values of MVIC-tests of type I behaviors. Table S4: Single values of MVIC-tests of type I behaviors.

## Abbreviations

1st Max.	first local maximum
1st Min.	first local minimum
1.96SD	1.96-fold standard deviation
AU	arbitrary units
BMI	body mass index
BV	blood volume
HIMA	holding isometric muscle action
IMA	isometric muscle action
M	arithmetic mean
MMG	mechanomyography
MVIC	maximal voluntary isometric contraction
NAD^+^/NADH	nicotine adenine dinucleotide (oxidized/reduced, H for hydrogen)
NAD(P)H	reduced form of nicotinamide adenine dinucleotide phosphate
Nox	NAD(P)H-oxidase
NI DIAdem^TM^	National Instruments DIAdem^TM^
NIRS	near infrared spectroscopy technique
NO	nitric oxide
O_2_^−^	superoxide anion
O2C	Oxygen To See (device, LEA Medizintechnik GmbH)
PIMA	pushing or pulling isometric muscle action
rHb	relative hemoglobin amount
RP	reversal point
SD	standard deviation
SvO_2_	capillary venous oxygen saturation of hemoglobin
tHb	total hemoglobin
THI	total hemoglobin index
TTF	time to task failure
