# Supplementary material for: Muscle Oxygenation Level Might Trigger the Regulation of Capillary Venous Blood Filling during Fatiguing Isometric Muscle Actions
Source: Diagnostics (Basel). 2021 Oct 23;11(11):1973. doi: 10.3390/diagnostics11111973 (PMC8621102; doi:10.3390/diagnostics11111973)
Supplement: Supplementary file 1 [file diagnostics-11-01973-s001.zip › diagnostics-1340737-supplementary.pdf]

diagnostics

MDPI

Article

Muscle oxygenation level might trigger the regulation of capillary venous blood filling during fatiguing isometric muscle actions

(Dech S, Bittmann FN and Schaefer LV)

Supplementary Materials

Table S1. Single values of fatiguing measurements of type I behavior.

IMA	subject no._arm	baseline value SvO_2 in %	baseline value rHb in AU	SvO_2 min. in %	TTF in s	SvO_2 SS mean in %	rHb SS mean in AU	extent of deoxygenation in pp (%)	extent of rHb decrease in AU (%)	time until min. of rHb before SS in s	time until SvO_2 SS in s	time until rHb SS in s	SvO_2 at RP_1 in %	SvO_2 at RP_2 in %	ρ between rHb & SvO_2
type I															
fatiguing HIMA	01_le	80.74	58.89	65.22	43.08	67.83	43.40	12.91 (10.42)	15.49 (9.12)	15.68	15.50	15.68	-	-	0.86
	06_le	67.54	48.63	60.23	70.48	61.40	47.76	6.14 (4.14)	0.87 (0.42)	9.90	17.13	9.90	-	-	0.40
	07_le	79.43	51.37	69.00	45.03	69.67	43.03	9.77 (7.76)	8.34 (4.29)	13.48	13.30	13.48	-	-	0.81
	17_le	80.40	58.75	68.15	48.93	69.09	45.36	11.30 (9.09)	13.39 (7.87)	15.93	15.93	15.93	-	-	0.99
	25_le	75.92	75.88	63.25	39.15	64.29	58.55	11.63 (8.83)	17.33 13.15)	11.70	13.68	11.70	-	-	0.57
	02_ri	81.10	66.18	63.00	37.20	63.29	43.71	17.81 (14.45)	22.47 14.87)	8.08	9.85	10.78	-	-	0.48
	07_ri	78.44	55.07	59.00	46.98	60.74	42.96	17.70 (13.89)	12.10 (6.66)	8.40	14.90	25.05	-	-	0.65
fatiguing PIMA	01_le	82.62	61.28	67.06	47.94	69.26	46.95	13.36 (11.04)	14.33 (8.78)	15.23	17.93	18.13	-	-	0.93
	06_le	73.91	53.99	60.21	48.12	61.03	47.98	12.87 (9.51)	6.01 (3.24)	8.53	14.85	8.53	-	-	0.19
	07_le	80.32	56.38	71.00	31.96	71.75	48.90	8.57 (6.88)	7.48 (4.22)	7.15	20.70	7.88	-	-	0.33
**M**		**78.04**	**58.64**	**64.61**	**45.88**	**65.84**	**46.86**	**12.21 (9.60)**	**11.78 (7.26)**	**11.41**	**15.38**	**13.70**	**-**	**-**	**0.74***
SD		4.50	7.84	4.13	10.26	4.13	4.68	3.67 (3.09)	6.24 (4.47)	3.44	2.91	5.22	-	-	0.61*

Abbreviations: AU = arbitrary units; IMA = isometric muscle action; HIMA = holding IMA; le = left; min. = minimum; M = arithmetic mean; MVIC = maximal voluntary isometric contraction; no. = number; PIMA = pulling IMA; pp = percent points; rHb = relative hemoglobin amount; ri = right; RP = reversal point; SD = standard deviation; SS = steady state; SvO_2 = capillary venous oxygen saturation of hemoglobin; TTF = time to task failure; ρ = Spearman's rank correlation coefficient.
* calculated by use of Fisher's Z-transformation

Table S2. Single values of fatiguing measurements of type II behavior.

IMA	subject no._arm	baseline value SvO_2 in %	baseline value rHb in AU	SvO_2 min. in %	TTF in s	SvO_2 SS mean in %	rHb SS mean in AU	extent of deoxy-genation in pp (%)	extent of rHb change in AU (%)	time until min. of rHb before SS in s	time until SvO_2 SS in s	time until rHb SS in s	SvO_2 at RP_1 in %	SvO_2 at RP_2 in %	ρ between rHb & SvO_2
							type II								
fatiguing HIMA	02_le	72.86	56.87	58.00	43.08	59.07	58.37	13.80 (10.05)	1.50 (0.88)	12.00	17.80	18.70	62.00	62.00	−0.81
	03_le	74.51	76.36	55.00	19.55	55.50	71.71	19.01 (14.16)	−4.64 (−3.33)	5.68	11.45	11.45	59.00	59.00	−0.41
	05_le	70.47	62.94	46.73	45.00	47.86	87.41	22.61 (15.94)	24.47 (21.39)	1.18	12.63	16.95	58.84	56.63	−0.74
	13_le	84.07	83.32	59.88	48.95	61.55	56.20	22.52 (18.93)	−27.12 (−15.24)	4.65	15.13	18.00	60.56	63.04	−0.91
	14_le	78.34	81.32	24.16	43.08	27.56	94.02	50.78 (39.78)	12.70 (11.94)	4.20	18.03	13.53	58.58	55.12	−0.48
	19_le	78.28	63.87	50.00	31.30	51.01	72.92	27.26 (21.34)	9.04 (6.59)	4.30	10.73	11.85	60.00	61.00	−0.91
	21_le	73.03	69.51	21.58	58.73	25.01	85.99	48.02 (35.07)	16.47 (14.17)	6.43	17.70	11.38	58.90	55.94	−0.18
	01_ri	64.01	65.92	50.72	45.03	51.98	83.97	12.03 (7.70)	18.05 (11.90)	4.83	24.93	23.13	58.70	58.36	−0.93
	03_ri	78.02	81.02	44.00	25.45	45.21	92.51	32.81 (25.60)	11.49 (10.63)	4.25	12.00	10.93	57.00	57.00	−0.83
	05_ri	62.27	75.68	37.93	39.15	42.36	106.82	19.90 (12.39)	31.14 (33.26)	6.03	11.40	14.13	55.08	55.50	−0.72
	06_ri	65.78	48.24	54.62	74.38	57.02	57.57	8.75 (5.76)	9.34 (5.37)	8.08	13.98	16.68	60.17	61.64	−0.97
	13_ri	62.38	56.58	48.00	47.00	49.92	72.61	12.46 (7.78)	16.03 (11.64)	15.55	15.13	16.75	59.00	61.00	−0.97
	14_ri	78.62	79.30	26.51	43.08	28.79	88.83	49.83 (39.18)	9.53 (8.47)	5.65	11.80	7.30	55.93	54.63	−0.46
	17_ri	72.72	57.62	55.38	41.13	56.72	65.11	16.00 (11.64)	7.49 (4.88)	7.18	13.90	13.90	61.76	61.84	−0.95
	19_ri	72.05	73.27	41.54	35.23	42.49	97.82	29.56 (21.30)	24.55 (24.01)	2.85	13.68	12.95	55.86	56.59	−0.46
	21_ri	76.89	84.91	43.80	39.13	45.69	88.80	31.20 (23.98)	3.89 (3.45)	5.65	11.63	13.43	55.56	56.51	−0.80
	24_ri	64.92	59.93	41.38	56.78	45.75	87.46	19.17 (12.44)	27.53 (24.08)	5.05	15.48	14.75	58.89	59.37	−0.97
fatiguing PIMA	02_le	80.32	56.38	59.00	47.00	59.00	64.34	21.32 (17.12)	7.96 (5.12)	4.05	13.65	12.00	62.00	63.00	−0.76
	03_le	78.08	79.23	40.00	48.93	43.38	84.29	34.70 (27.09)	5.06 (4.26)	11.83	14.83	18.45	58.00	55.00	−0.55
	05_le	74.91	73.18	50.83	29.38	51.77	82.00	23.14 (17.33)	8.83 (7.24)	6.38	8.10	8.83	57.27	58.25	−0.63
	01_ri	73.99	76.63	54.04	48.93	55.52	75.94	18.47 (13.66)	−0.68 (−0.52)	9.55	25.20	23.38	58.50	60.15	−0.71
	02_ri	69.81	55.88	60.00	58.70	60.70	58.51	9.12 (6.37)	2.62 (1.53)	6.23	16.18	23.88	62.00	62.00	−0.87
	03_ri	78.29	76.83	44.00	54.80	47.81	83.69	30.48 (23.86)	6.87 (5.75)	7.90	17.15	16.25	59.00	60.00	−0.96
	05_ri	66.90	78.99	41.09	33.28	41.64	100.89	25.25 (16.89)	21.89 (22.09)	5.98	19.55	16.63	54.60	56.40	−0.79
	06_ri	79.64	59.07	56.19	66.55	58.26	54.49	21.38 (17.03)	−4.58 (−2.50)	14.03	16.40	17.30	60.30	61.80	−0.87

	07_ri	74.25	49.51	57.00	52.85	58.03	43.18	16.22 (12.04)	−6.32 (−2.73)	8.65	14.50	9.10	60.00	60.00	−0.18
**M**		**73.28**	**68.55**	**46.98**	**45.25**	**48.83**	**77.52**	**24.45 (18.25)**	**8.96 (8.24)**	**6.85**	**15.11**	**15.06**	**58.75**	**58.91**	**−0.81***
SD		5.97	11.21	10.79	12.40	10.09	16.35	11.59 (9.35)	12.39 (10.48)	3.39	3.95	4.35	2.14	2.72	0.52*

Note: Abbreviations can be found on the first page.

Table S3. Single values of MVIC-tests of type I behaviors.

**IMA**	**subject no._arm trial no.**	**baseline value SvO_2 in %**	**baseline value rHb in AU**	**SvO_2 min. in %**	**TTF in s**	**SvO_2 SS mean in %**	**rHb SS mean in AU**	**extent of deoxygenation in pp (%)**	**extent of rHb decrease in AU (%)**	**time until min. of rHb before SS in s**	**time until SvO_2 SS in s**	**time until rHb SS in s**	**SvO_2 at RP_1 in %**	**SvO_2 at RP_2 in %**
								type I						
MVIC-test PIMA	01_le_1	81.35	57.73	70.13	~4s	-	-	11.22 (13.79)	10.63 (18.42)	-	-	-	-	-
	01_le_2	77.14	53.64	64.6	~4s	-	-	12.54 (16.26)	12.53 (23.36)	-	-	-	-	-
	02_le_1	71.52	54.00	62.29	~4s	-	-	9.23 (12.91)	11.07 (20.50)	-	-	-	-	-
	02_le_2	64.51	54.79	61.64	~4s	-	-	2.87 (4.45)	10.15 (18.52)	-	-	-	-	-
	06_le_1	67.12	49.47	61.26	~4s	-	-	5.86 (8.73)	5.79 (11.70)	-	-	-	-	-
	06_le_2	66.18	51.65	60.36	~4s	-	-	5.82 (8.80)	6.79 (13.14)	-	-	-	-	-
	07_le_1	76.32	46.44	71.29	~4s	-	-	5.03 (6.59)	4.87 (10.49)	-	-	-	-	-
	07_le_2	77.35	48.61	71	~4s	-	-	6.35 (8.21)	6.98 (14.35)	-	-	-	-	-
	01_ri_1	69.48	64.52	57.43	~4s	-	-	12.05 (17.34)	11.68 (18.10)	-	-	-	-	-
	02_ri_1	67.91	51.31	62.29	~4s	-	-	5.62 (8.28)	5.02 (9.79)	-	-	-	-	-
	02_ri_2	70.36	54.58	62.00	~4s	-	-	8.36 (11.88)	7.94 (14.55)	-	-	-	-	-
	06_ri_1	66.66	45.19	59.76	~4s	-	-	6.90 (10.36)	5.26 (11.64)	-	-	-	-	-
	06_ri_2	68.53	48.00	60.02	~4s	-	-	8.51 (12.42)	7.50 (15.63)	-	-	-	-	-
	07_ri_1	73.95	46.93	60.00	~4s	-	-	13.95 (18.86)	12.93 (27.56)	-	-	-	-	-
	07_ri_2	72.16	46.21	57.64	~4s	-	-	14.52 (20.13)	8.92 (19.30)	-	-	-	-	-
**M**		**71.37**	**51.54**	**62.78**	**-**	**-**	**-**	**8.59 (11.93)**	**8.54 (16.47)**	**-**	**-**	**-**	**-**	**-**
SD		4.92	5.20	4.53	-	-	-	3.55 (4.64)	2.80 (5.01)	-	-	-	-	-

Note: Abbreviations can be found on the first page.

Table S4. Single values of MVIC-tests of type II behaviors.

IMA.	subject no._arm trial no.	baseline value SvO_2 in %	baseline value rHb in AU	SvO_2 min. in %	TTF in s	SvO_2 SS mean in %	rHb SS mean in AU	extent of deoxygenation in pp (%)	extent of rHb change in AU (%)	time until min. of rHb before SS in s	time until SvO_2 SS in s	time until rHb SS in s	SvO_2 at RP_1 in %	SvO_2 at RP_2 in %
type II														
MVIC-test PIMA	03_le_1	69.55	71.04	50.64	~4s	-	-	18.91 (27.19)	2.68 (3.78)	-	-	-	57.19	58.71
	03_le_2	76.22	76.14	57.29	~4s	-	-	18.93 (24.84)	−14.07 (−18.48)	-	-	-	57.46	58.07
	05_le_1	72.89	64.51	53.90	~4s	-	-	18.99 (26.05)	−1.48 (−2.29)	-	-	-	61.69	58.42
	05_le_2	65.74	61.05	50.24	~4s	-	-	15.50 (23.58)	13.10 (21.45)	-	-	-	59.32	58.43
	01_ri_2	63.09	65.87	53.63	~4s	-	-	9.46 (14.99)	9.23 (14.01)	-	-	-	59.12	58.47
	03_ri_1	70.03	67.19	50.64	~4s	-	-	19.39 (27.69)	5.48 (8.16)	-	-	-	57.21	57.93
	03_ri_2	69.67	67.33	49.29	~4s	-	-	20.38 (29.25)	9.45 (14.04)	-	-	-	57.50	58.93
	05_ri_1	64.06	70.48	48.37	~4s	-	-	15.69 (24.50)	7.43 (10.54)	-	-	-	57.48	56.02
	05_ri_2	65.32	73.07	50.19	~4s	-	-	15.13 (23.17)	3.83 (5.24)	-	-	-	56.16	55.38
**M**		**68.51**	**68.52**	**51.58**	**-**	**-**	**-**	**16.93 (24.58)**	**3.96 (6.27)**	**-**	**-**	**-**	**58.13**	**57.82**
SD		4.34	4.62	2.81	-	-	-	3.41 (4.12)	8.00 (11.54)	-	-	-	1.66	1.25

Note: Abbreviations can be found on the first page.
